# 

**Published:** 1886-10-23

**Authors:** 


### The Hospital.

An Institution, Family, and Parochial Journal of Hospitals, Asylums, and all Agencies for the Care of the Sick, Criticism and News.

SATURDAY, OCTOBER 23RD, 1886.

### SPECIAL NOTICES.

A CONVERSAZIONE of the friends and supporters of hospitals and asylums throughout the country, will be held at the rooms of the Medical Society of London, Chandos Street, Cavendish Square, on the evening of Wednesday, November 17th, 1886. Arrangements are being made for an exhibition of appliances used in the treatment of the sick, and any suggestions or contributions to this exhibition will be gladly received by the Hon. Secs., Hospitals Association, Norfolk House, Norfolk Street, London, W.C. Sir Andrew Clark, Bart., M.D., F.R.S., will receive the guests, as President of the Hospitals Association, all members of which will be entitled to be present.

IN reply to many inquiries, we have to state that arrangements have now been made to publish an In and Out-Patients' Hospital Diary each week. An In-Patients' Diary will be found on page 67 of the current number, in continuance of that for Out-Patients on page 34.

### NOTES AND NEWS.

### "The Hospital" Prize Competitions.

We offer for competition among our readers money prizes, classified under the subjoined heads. We are anxious that this feature of The Hospital shall prove of interest to all classes of readers, and the grouping of the competitions has been arranged solely with this end in view.

All competitors who send in papers must strictly abide by the following

**Conditions.**

I. Every paper sent in must be clearly written, and one side only must be used. All competitors must mark at the head of their papers the number of their competition.

II. Each paper must be signed by the author with his or her real name and address. A *nom de plume* may be added, if the writer does not desire to be referred to by us by his real name. In the case of all prize-winners, however, the real name and address will be published.

III. All communications relating to prize competitions should be addressed to "The Prize Editor, The Hospital Office, Norfolk House, Norfolk Street, Strand, W.C." Competitors are requested not to include in letters, etc., to the Prize Editor communications on matters of other business. All letters must be fully prepaid.

IV. The Prize Editor of The Hospital is the sole judge of the competitions, and his decision is in all cases final and without appeal.

**V. All competitions must be received at "The Hospital" Office by the dates stated, failing which they cannot be dealt with. Every Reply must be accompanied by the Prize Competition "Coupon" which appears on another page.**

VI. The Prize Editor reserves the right to publish any paper sent in, whether it receives a prize or not. He does not hold himself responsible for any MSS. sent, nor can he undertake to return rejected competitions.

VII. No competition must exceed two columns in length, except the Story competition, which must not exceed six columns.

The One Guinea Prize.

A prize of *One Guinea* will be offered every fortnight to all readers of The Hospital for the best *Story* or narrative based upon hospital, asylum, or student life, or upon any incident connected with the professions of medicine or nursing. The Prize Competition will be published in The Hospital.

Competitions must be received by Saturday, 30th October. Envelopes to be marked in left-hand corner "Story."

The Half-Guinea Prize.

A prize of *Half-a-Guinea* is offered fortnightly to all readers, for the best *Anecdote* or *Personal Reminiscence* of any interesting circumstance which may have come under their observation while at or attending any hospital.

Competitions must be received by Saturday, 30th October. Envelopes to be marked in left-hand corner, "Anecdote" or "Reminiscence."

The Five Shillings Prize.

A Prize of *Five Shillings* is offered fortnightly to all readers of The Hospital for the best *Item of News* suitable for publication in this Journal.

Competitions must be received by Saturday, 30th October. Envelopes to be marked in left-hand corner, "News."

The Nine Pounds Quarterly Prizes.

Prizes of *Five, Three, and One Pound* will be awarded on 2nd January 1887, to the three persons who shall have obtained during the months of October, November, and December, 1886, the largest number of subscribers to The Hospital. Order Forms, for subscribers to fill up and sign, may be obtained on application to the publishers, 30 and 32, Sardinia Street, Lincoln's Inn Fields, London, W.C. The Prize Editor must be informed by competitors from time to time of the names and addresses of new subscribers they procure. Names will be received, up to and including Saturday, 23rd December, 1886.

**Results of the First Competition.**

The One Guinea Prize.

No *Story* of sufficient merit having been received, we regret that we have not been able on this occasion to award the One Guinea Prize.

The Half-Guinea Prize.

We have much pleasure in awarding this prize to a Cottage Hospital Nurse, *Miss Sharpley*, of the Boston Cottage Hospital, Boston, Lincolnshire, for her *Reminis-*

*cence*, entitled "Willie, a True Story." We shall probably publish the *Reminiscence* in THE HOSPITAL.

THE FIVE SHILLINGS PRIZE, for the best *Item of News*, for which there were numerous competitors, has been given to *Mrs. Myers*, Duningwell, Broughton-in-Furness. The *Item* was published in THE HOSPITAL on the 16th inst.

### THE CHILDREN'S CORNER.

THE numerous assurances of which I have been the recipient during the past few days confirm the hope which I ventured to express in the initial number of THE HOSPITAL, that the Children's Corner would prove a source of amusement to my little readers. "Hetty" says that she is going to introduce the paper to all her young friends, and adds: "I am sure they will be as delighted with the Children's Corner as I have been." I thank "Hetty" for her thoughtful kindness, and I hope it will produce many imitators. Master George Collins says he has a lot of puzzles if I would like them. I shall be very glad to see them, if my correspondent will send them on.

### OUR PASTIMES.

**Children's Quarterly Prize Competition.**

| | |
|---|---|
| Began | 2nd Oct., 1886 |
| Ends | 25th Dec., 1886 |

PRIZES.

| | |
|---|---|
| 1st | Thirty Shillings |
| 2nd | Twenty " |
| 3rd | Ten " |
| 4th | Five " |

will be awarded to the four competitors, who shall have sent in, during the thirteen weeks, the largest number of correct answers to our pastimes.

**Fourth Competition.**

No. 16. Spell "foe" without using an "f," an "o," or an "e."

No. 17. Who can guess this word of eight letters?—

I am in hat, but not in cap,
I am in strop, but not in strap,
I am in sugar, but not in tart,
I am in weapon, but not in dart,
I am in iris, but not in eye,
I am in pastry, but not in pie,
I am in near, but not in nigh,
I am in latchet, but not in tie.

No. 18. There is a word beginning with "s," and ending with "s," and, though it contains only six letters, is yet the longest in the English language. What is the word?

No. 19. The following letters compose a common English word. What is it?—s s s s s s e e e e n n l.

No. 20. Who is the first man mentioned in the Bible?

**Results of Second Competition.**

No. 5. I am afraid this was really too easy, for nearly all my little readers have guessed the answer, "E."

No. 6. This puzzle, which is, I believe, of American origin, affords plenty of play for the exercise of one's ingenuity. *East to West* is easily transformed, thus, *East, vast, vest, West*, or *East, wast, West;* but *Skull to Greek* is far more difficult, owing to the awkward combinations of *Sk* and *Gr*. It can, however, be done thus: *Skull, skill, swill, twill, trill, troll, trowl, growl, grown, groan, groat, great, greet, Greek*. But there are many other possible ways. "Retsibsi" has alone accomplished the task. "Alsie" has erred in not changing the *corresponding letter*, in each mutation, thus she makes *Skull* change to *kills*, instead of to *skill*.

No. 7. The English of my Latin (!) reads:

I say, Billy, here's a go,
Forty 'buses in a row.

Nearly everyone has found it out.

No. 8. "Alsie" comes off with sole honours here, for no one else has guessed the shoeblack riddle right. Master Tynget, and "Retsibsi" have ingeniously suggested "Blackie" (Black'ye), the publisher, while "Florrie" thinks "Day and Martin," but surely, if you hailed a shoeblack from the other side of the road, would you not more probably say, "Crosse and Black-well!"

No. 9. The only correct answer here comes from "T. K." There are, I believe, no words, other than *abstemious* and *facetious* which give the five vowels (and no more), in their alphabetical order. "Alsie" gives *adventitious*, but here are two "i's."

My little friends have made an excellent start, and I hope they will continue so energetic. You know a race which is keenly contested to the finish always affords the most interest and excitement.

All right—None.
Four right—Filomel.
Three right—T. K., Little Mary, Alsie, Retsibsi.
Two right—C. Tynget, Hetty.
One right—Florrie, Charlie Powell, Diosota, and Ubique.

Anwers, having the actual name and address (to which a pseudonym may be added for publication), must have "The Children's" Coupon attached (see advertisement pages), addressed to the "PUZZLE EDITOR, THE HOSPITAL, Norfolk House, Norfolk Street, Strand, W.C.," not later than Thursday, 28th October. Results will appear in our issue of the 6th November.

### "THE HOSPITAL" CHARITY BOX.

OUR "Charity Box" is intended to afford a little practical help to the hospitals. Each week we propose a word for "anatomical dissection," and the first 150 competitors who extract the largest number of words from it receive the following prizes:—

| | |
|---|---|
| 1st | One Guinea. |
| 2nd | Half a Guinea. |
| 3rd | Five Shillings. |

The above Prizes will be repeated for every additional 150 competitors who may enter each week.

THIRD COMPETITION.

Compose the largest number of words you can from the name—

SYDENHAM.

*Observe:* Proper names, abbreviations, foreign and obsolete words, and repetitions are barred.

Write only on one side of the paper, and total your words.

Append your real name and address.

All replies, addressed "Charity Box," THE HOSPITAL, Norfolk House, Norfolk Street, W.C., must arrive by Friday, October 29th. Results will appear Saturday, Nov. 6th.

*Every Competitor is required to cut out and enclose with his list the "Charity Box" Coupon* (see advertisement pages), *and a shilling entrance fee* (postal orders only).

The proceeds resulting from each "Charity Box" competition will be funded until the end of December 1886. The total sum available for distribution, and the names of the hospitals selected for donations, will appear on Saturday, January 8, 1887. The distribution of the proceeds will be at the Editor's sole discretion.

RESULT OF SECOND COMPETITION.

The "Linacre" Competition has resulted as follows:—

| | Score. |
|---|---|
| *Miss M. Walmesley*, 7, Scarsdale Terrace, Kensington, W. | 120—1st Prize. |
| *Miss Alice Clift*, Haroldene, Vermont Road, Upper Norwood | 105—2nd Prize. |
| *Mr. J. Kent*, Connaught Mansions, Westminster, S.W. | 94—3rd Prize. |

We must again ask competitors not to extend their lists by the inclusion of barred words, as their excision gives unnecessary trouble. This has reduced totals in several cases.

### NOTICE TO CORRESPONDENTS.

All correspondence must be written on one side of the paper only.

We cannot undertake to return MSS. not used.

**Communications**, whether intended for insertion or for private information, must be authenticated by the names and addresses of the writers, not necessarily for publication.

**Local papers** containing reports or news paragraphs **should be marked.**

It is especially requested that early intelligence of **local events of interest**, or which it may be thought desirable to bring under notice, may be sent direct to the Editor.

Communications respecting editorial matters should be addressed to the Editor, Norfolk House, Norfolk Street, Strand, London, W.C.

### BUSINESS COMMUNICATIONS AND ADVERTISEMENTS.

All communications concerning business matters, non-delivery of THE HOSPITAL, etc., should be addressed to the Manager, at the Publishing Offices, 30 and 32, Sardinia Street, Lincoln's Inn Fields, London, W.C.

**Cheap Advertisements** of the "Wanted" class, including those for situations, of Twenty-four Words or under, are charged 1*s*. an insertion, or three insertions for 2*s*. 6*d*., but must be paid for in advance. Each additional line of Eight Words is 4*d*.

**Employers requiring assistants** are charged for one insertion of Twenty-four Words or under, 2*s*. 6*d*., or 6*s*. for three insertions. Each additional line of Eight Words is 1*s*.

**Advertisements** for insertion in the weekly issue of THE HOSPITAL **must be delivered** at the Publishing Offices **not later than 11 a.m. on Wednesdays.**

Cheques and Post Office Orders to be made payable to Messrs. WHITING & Co. Subscribers and Advertisers are requested **not to send stamps.**

### The In-patients' Hospital Diary.

The following Table gives the terms of admission, the names of the Physicians and Surgeons, and the days and hours of their attendance upon the In-patients at all the General Hospitals in the Metropolis, and at the Special Hospitals, in alphabetical order of Diseases, up to and including half the Ophthalmic Hospitals. The rest of the Hospitals will appear next week.

### I.—GENERAL HOSPITALS.

| Hospitals and Terms of Admission. | Medical Officers, and Days and Hours of Attendance. | |
|---|---|---|
| | Physicians. | Surgeons. |
| Charing Cross Hospital, West Strand, W.C.<br>Cases of accident or emergency are immediately admitted as in-patients. Subscriber's letter expected in other cases | Dr. Pollock<br>„ Green<br>„ Bruce<br>Daily, 1.30 and 2; W., 12 | Mr. Barwell<br>„ Bellamy<br>„ Bloxam<br>S., 10; M., Tu., Th., F., 1.30; M., Tu., W., Th., F., 2 |
| German Hospital, Dalston Lane, E.<br>Free, or by Governor's letter | Dr. H. Weber<br>„ H. Port<br>M., Th., 2; Tu., F., 9 | Dr. Lichtenberg<br>„ A. Burger<br>W., S., 2 |
| Great Northern Central Hospital, Caledonian Road, N.<br>Free to all | Dr. Cholmeley<br>„ Cook<br>„ Burnett<br>M., Tu., Th., 2; S., 10 | Mr. W. Adams<br>„ W. Watson<br>„ J. Macready<br>M., Tu., Th., F., 2 |
| Guy's Hospital, St. Thomas's Street, S.E.<br>Admission free | Dr. Pavy<br>„ Pye-Smith<br>„ Taylor<br>M., W., F., 12.30; M., Tu., Th., F., S., 1.30 | Mr. Bryant<br>„ Durham<br>„ Howse<br>„ D. Colley<br>Daily, 1.30; Tu., 12 |
| King's College Hospital, Portugal Street, W.C.<br>By Governors' letters; urgent cases free at all times | Dr. Beale<br>„ Duffin<br>„ I. B. Yeo<br>„ Dalton<br>Tu., W., F., 1.30; Tu., W., F., S., 2 | Mr. Wood<br>Sir J. Lister<br>Mr. H. Smith<br>„ Barrow<br>Daily, 1.30 |
| London Hospital, Whitechapel Road, E.<br>Admission free | Dr. Mackenzie<br>„ Down<br>„ H. Jackson<br>„ Sutton<br>„ Fenwick<br>W., 9.30; Tu., W., F., S., 1.30; Daily, 2 p.m. | Mr. Rivington<br>„ Tay<br>„ McCarthy<br>„ Treves<br>„ J. Sutton<br>M., Tu., Th., F., S., 2; M., Tu., W., F., S., 1.30 |
| London Temperance Hospital, Hampstead Road, N.W.<br>For treatment without alcohol | Dr. J. Edmunds<br>„ R. J. Lee | Mr. A. P. Gould<br>„ G. C. Wilkin |
| Metropolitan Free Hospital, Kingsland Road, N.<br>Admission free | Dr. Drysdale<br>„ J. G. Dudley<br>„ H. Fotherly<br>„ H. H. Tooth<br>„ A. Haig<br>„ A. Davies<br>Daily, 9 and 12 | Mr. E. Chance<br>„ A. Goodsall<br>„ Walsham<br>„ A. Bowlby<br>„ S. Page<br>Daily, 9 |
| Middlesex Hospital, Berners Street, W.<br>Admission free | Dr. Cayley<br>„ Coupland<br>„ D. Powell<br>„ Finlay<br>Daily, 1.30; Tu., F., 3.30 | Mr. Hulke<br>„ Lawson<br>„ Morris<br>M., Tu., Th., F., 1.30; W., S., 9; M., Th., 1.15 |
| North-West London Hospital, 18, Kentish Town Road, N.W.<br>By subscribers' free tickets | Dr. W. C. Hood<br>„ J. Shaw<br>„ E. Edwardes<br>„ Wolfenden<br>Daily, 1.30 | Mr. F. Durham<br>„ M. Collier<br>„ C. Jennings<br>„ J. Black<br>Daily, 1.30 |
| Poplar Hospital, 303, East India Dock Road, E.<br>Free to accidents and emergencies | .... | Mr. F. Corner<br>„ Brownfield |
| Royal Free Hospital, Gray's Inn Road, W.C.<br>Admission free | Dr. Cockle<br>„ S. West<br>„ Sainsbury<br>Daily, 1 | Mr. F. Grant<br>„ W. Rose<br>„ A. Barrow<br>Daily, 1 |
| St. Bartholomew's Hospital, Smithfield<br>Admission free | Dr. Andrew<br>„ Church<br>„ Gee<br>Sir D. Duckworth<br>Daily, 11 and 1.30 | Mr. Savory<br>„ T. Smith<br>„ Willett<br>„ Langton<br>„ M. Baker<br>Daily, 12.30 1.30 |
| St. George's Hospital, Hyde Pk. Corner, S.W.<br>By Governor's letter | Dr. Wadham<br>„ Dickinson<br>„ Whipham<br>„ Cavafy<br>Daily, 1 p.m. | Mr. Holmes<br>„ Rouse<br>„ Haward<br>Daily, 1 and 1.30 |
| St. Mary's Hospital, Cambridge Place, Paddington, W.<br>By Governor's letter | Sir E. Sieveking<br>Dr. Broadbent<br>„ Cheadle<br>S., 9.30; W., 1.45; W., S., 1.30.; M., Tu., Th., F., 1.45 | Mr. Norton<br>„ Owen<br>„ H. Page<br>Daily, 1.30 |
| St. Thomas's Hospital, Westminster Bridge Road, S.E.<br>Vaccination on Wednesday at 11.30 | Dr. Bristowe<br>„ Stone<br>„ Ord<br>„ J. Harley<br>Daily, 1.30; M., Tu., Th., F., 2 | Mr. S. Jones<br>„ Croft<br>Sir W. Mac Cormac<br>Daily, 1.30; M., Tu., Th., F., 2 |
| Seamen's Hospital (late Dreadnought), Greenwich, S.E.<br>Entirely free to seamen, and to all accident cases | Dr. F. Curnow<br>„ H. Griffiths | Mr. W. J. Smith<br>„ G. R. Turner |
| Tottenham Training Hospital, Tottenham, N. | Dr. Rasch<br>Tu., F., 3 to 5 | Dr. Lichtenberg<br>Mr. E. H. May<br>M., Th., 3 to 5 |
| University College Hospital, Gower St., W.C.<br>By Governors' letters. Accidents and emergencies free | Dr. Wilson Fox<br>„ Ringer<br>„ Bastian<br>„ F. Roberts<br>Daily, 1 and 2 | Mr. B. Hill<br>„ C. Heath<br>„ M. Beck<br>Daily, 1 and 2 |
| West London Hospital, Hammersmith Road, W.<br>By Governors' letters. New cases must attend at 1.30 | Dr. G. Rogers<br>„ D. W. Hood<br>„ F. Drewitt<br>„ S. Taylor | Mr. C. Keetley<br>„ F. Edwards<br>„ W. Clarke |
| Westminster Hospital, Broad Sanctuary, S.W.<br>By Governors' letters. Accidents and urgent cases free | Dr. Sturges<br>„ Allchin<br>„ Donkin<br>Daily, 1.30 | Mr. Cowell<br>„ Davy<br>„ Macnamara<br>Daily, 1.30; S., 9.30 |

### II.—SPECIAL HOSPITALS.

### CANCER.

| Hospitals and Terms of Admission. | Medical Officers, and Days and Hours of Attendance. | |
|---|---|---|
| | Physicians. | Surgeons. |
| Cancer Hospital, Brompton, S.W.<br>Entirely free | Dr. A. Marsden<br>„ H. Snow<br>„ F. Purcell<br>Daily, 2 | Mr. F. B. Jessett<br>„ W. Elam<br>„ C. Stonham<br>„ C. Jennings<br>Daily, 2 |
| Middlesex Hospital, Berners Street, W. Free. See p. 16 | .... | Mr. Morris<br>Th., 1.30 |
| St. Saviour's Hospital, Osnaburg Street, N.W.<br>By Governors' letters. | Dr. A. Kennedy | Daily, 10.30 and 4 |

### CHILDREN'S DISEASES.

| Hospitals and Terms of Admission. | Medical Officers, and Days and Hours of Attendance. | |
|---|---|---|
| | Physicians. | Surgeons. |
| Alexandra Hosp. for Children, 18, Queen's Sq., W.C.<br>For hip disease. | .... | Mr. H. Marsh<br>„ A. Bowlby |
| All Saints' Children's Hospital, 4, Margaret St., W.<br>For chronic joint diseases. Children under 10 | .... | Mr. N. Smith |
| Belgrave Osp. for Children, 79, Gloucester Street, Pimlico.<br>By Governors' letters | Dr. W. Hope<br>„ W. Ewart<br>M., Tu., Th., F., 9 | Mr. C. T. Dent<br>„ Margerison<br>M., Tu., Th., F., 9 |

### CHILDREN'S DISEASES—*continued.*

| Hospitals and Terms of Admission. | Medical Officers, and Days and Hours of Attendance. | |
|---|---|---|
| | Physicians. | Surgeons. |
| Cheyne Hospital for Sick and Incurable Children, 46, Cheyne Walk, Chelsea, S.W.<br>In-patients only | Dr. G. V. Poore | Mr. J. Macready<br>„ J. Bartlett |
| East London Hospital for Children and Dispensary for Women, Glamis Road, Shadwell, E.<br>By Governor's letter. Urgent accident cases free | Dr. E. Smith<br>„ H. Donkin<br>„ H. Crocker<br>„ J. A. Coutts<br>„ D. Williams | Mr. A. Cæsar<br>„ R. Parker<br>„ L. A. Dunn |
| Evelina Hospital for Sick Children, Southwark Bridge Road, S.E.<br>Free, but Governor's letter takes precedence | Dr. F. Taylor<br>„ J. Goodhart | Mr. H. Howse<br>„ R. C. Lucas |
| Grosvenor Hospital for Women and Children, Vincent Sq., Westminster, S.W.<br>By Subscriber's letter or payment | Dr. A. Smythe<br>„ R. Gibbons<br>„ Steavenson | Mr. R. Parker |
| Hospital for Sick Children, 49, Great Ormond Street, W.C.<br>By Governors' letters. Other cases investigated. Patients under 12 | Dr. W. Cheadle<br>„ Sturges<br>„ Barlow | Mr. H. Marsh<br>„ E. Owen |
| King's College Hospital.<br>By Governor's letter. See p. 16 | Dr. T. C. Hayes | W., F., 1 |
| North-Eastern Hospital for Children, Hackney Road, E.<br>By subscriber's free ticket; or by payment of 4*d.* on admission, and 3*d.* per work | Dr. W. Cayley<br>„ F. Turner<br>„ C. Semple<br>„ W. Pasteur | Mr. Waren Tay<br>„ R. Godlee |
| Paddington Green Children's Hospital, Paddington Green, W.<br>Admission free | Dr. T. Brunton<br>„ L. Ogilvie<br>„ G. Laycock<br>„ S. Phillips | Mr. W. Cheyne<br>„ S. Boyd |
| Royal Hospital for Children and Women, Waterloo Bridge Road, S.E.<br>Boys over 12 not admissible | Dr. W. Duncan<br>Daily | Mr. H. Jacobson<br>M., Th. |
| St. Thomas's Hospital, Westminster Bridge Road. See p. 16 | Dr. Cory | W., S., 1.30 |
| Samaritan Free Hospital for Women and Children, Lower Seymour Street, W.; Branch, 1, Dorset St., Manchester Sq., W.<br>Governor's letter or card is necessary if the disease of the applicant is *not* peculiar to her sex | Dr. M. Prickett<br>„ A. Routh<br>„ W. H. Day<br>„ P. Boulton | Mr. Bantock<br>„ Thornton<br>„ Meredith |
| Victoria Hosp. for Children, Queen's Road, Chelsea, S.W.<br>Age of boys, 2 to 12 years; age of girls, 2 to 16 | Dr. J. Evans<br>„ Ridge-Jones | Mr. Pick<br>„ Clutton |

### CONSUMPTION AND DISEASES OF THE CHEST.

| Hospitals and Terms of Admission. | Physicians. | Surgeons. |
|---|---|---|
| City of London Hospital for Diseases of the Chest, Victoria Park, E.<br>By subscriber's letter available for 6 weeks. Examinations, S. | Dr. J. Thorowgood<br>„ E. Smith<br>„ J. Fothergill<br>„ S. West<br>„ G. A. Heron<br>„ V. D. Harris | } Daily, 2 |
| Hospital for Consumption, Brompton, S.W.<br>By subscriber's letter | Dr. Thompson<br>„ C. Williams<br>„ R. Powell<br>„ J. Tatham<br>„ Thompson<br>„ F. Roberts<br>Daily, 2 to 4 | Mr. R. Godlee<br>F., and when wanted |
| Infirmary for Consumption, 26, Margaret St., Cavendish Sq., W.<br>By subscriber's letter available for 8 weeks | Dr. C. Torry<br>„ Jagielaki<br>„ J. Barratt<br>Daily, 2 | Mr. F. Carr-Beard<br>Daily, 2 |
| North London Hospital for Consumption, Mount Vernon, Hampstead, N.<br>Admission by subscriber's letter available for 6 weeks | Dr. A. Evershed<br>„ E. Haward<br>„ J. Shaw<br>„ B. O'Connor<br>„ G. Pidcock<br>„ S. Taylor<br>„ J. Cassiday<br>„ G. Squire<br>„ W. Boulting | } M. and S. 1; Tu., W., Th., F., 2 |
| Royal Hospital for Diseases of the Chest, City Road, E.C.<br>By Governor's letter | Dr. O. Browne<br>„ A. Davies<br>„ Hobershon<br>„ E. Stewart | Mr. Pearce-Gould |

### CONSUMPTION & DISEASES OF THE CHEST—*continued.*

| Hospitals and Terms of Admission. | Medical Officers, and Days and Hours of Attendance. | |
|---|---|---|
| | Physicians. | Surgeons. |
| Royal National Hospital for Consumption and Diseases of the Chest, Ventnor. The London offices are at 34, Craven St., Strand.<br>Admit only incipient cases | Dr. J. Cockle<br>„ H. Weber<br>„ H. Port<br>„ Thorowgood<br>„ A. Davis<br>„ J. Coghill<br>„ Robertson | Mr. Whitehead<br>„ Williamson |

### EAR CASES.

| | | |
|---|---|---|
| Central London Throat and Ear Hospital, Gray's Inn Road, W.C.<br>Free, but when able, a small payment is expected | Dr. J. Grant | Mr. P. S. Jakins<br>„ T. W. Jones |
| Charing Cross Hospital, West Strand, W.C. | F., 9 | Mr. Cantlie |
| Great Northern Central Hospital. See p. 16 | .... | Mr. J. E. Cumberbatch |
| Guy's Hospital, St. Thomas's St., S.E. See p. 16 | Tu., F., 12.30 | Mr. Purves |
| King's College Hospital, Portugal Street, W.C. See p. 16 | Th., 2.30 | Mr. Pritchard |
| London Hospital, Whitechapel Road, E. See p. 16 | Dr. Woakes<br>S., 9.30 | Mr. Hovell<br>S., 9.30 |
| Metropolitan Ear and Throat Infirmary, 13, Howland St., W.<br>By subscribers' letters, or by payment; free to very poor | Dr. Pickett<br>„ D. Nesbitt | .... |
| Middlesex Hospital, Berners Street, W. See p. 16 | Tu., 9 | Mr. Hensman |
| Municipal Throat and Ear Infirmary, 266, City Road, E.C.<br>Free to necessitous; or by payment | Dr. G. Holmes<br>„ J. A. Hatch | .... |
| National Hospital for the Paralysed and Epileptic, Queen Square, W.C. | .... | Mr. A. Cumberbatch |
| Royal Ear Hospital, Frith Street, Soho, W.<br>Admission by subscriber's ticket; free to the very poor | Dr. U. Pritchard<br>„ F. Matheson | .... |
| St. Bartholomew's Hospital, Smithfield, E.C. See p. 16 | Mr. Cumberbatch | Tu., F., 2 |
| St. George's Hospital, Hyde Park, W. See p. 16 | Tu., 2 | Sir W. B. Dalby |
| St. Mary's Hospital, Paddington.<br>By Governors' letters. See p. 16 | W., S., 9.30 | Mr. G. P. Field |
| St. Thomas's Hospital, Westminster Bdg. Rd., S.E. See p. 16 | M., 1.30 | Mr. Clutton |
| University College Hospital.<br>By Governors' letters. See p. 16 | S., 1.30 | Mr. Barker |
| Westminster Hospital.<br>By Governors' letters. See p. 16 | Tu., F., 9 | Mr. Barrow |

### EYE DISEASES.

| | | |
|---|---|---|
| Central London Ophthalmic Hospital, Gray's Inn Rd., W.C.<br>Admission free | Daily, 1 | Mr. T. Archer<br>„ G. Abbott<br>„ Charnley<br>„ W. Jessop<br>„ E. Clarke<br>„ J. R. Kemp |
| Evelina Hospital for Sick Children, Southwark Bridge Road, S.E. | .... | Dr. W. Brailey |
| Great Northern Central Hospital, Caledonian Rd., N. Free | Tu., F., 9.30 | Mr. Hutchinson, Jnr. |
| Guy's Hospital, St. Thomas's Street, S.E. See p. 16 | Tu., F., 12.30 | Mr. Higgins<br>Dr. Brailey |
| Hospital for Sick Children, 49, Great Ormond Street, W.C. | Tu., 2 | Mr. R. Gunn |
| King's College Hospital.<br>By Governors' letters. See p. 16 | M., W., Th., 1.30 | Mr. McHardy |
| London Hospital.<br>By Governors' letters. See p. 16 | M., W., 1.30;<br>Tu., S., 9 | Mr. W. Tay<br>„ Eve |
| Middlesex Hospital.<br>By Governors' letters. See p. 16 | Tu., F., 10 | Mr. Lang |
| National Hospital for the Paralysed and Epileptic, Queen's Sq., W.C. | .... | Mr. R. Carter<br>„ M. Gunn |
| North-West London Hospital,<br>By Governors' letters. See p. 16 | Dr. W. Collins | F., 9.30 |
| Paddington Green Children's Hospital. Free. See p. 32 | Th., 9 | Mr. W. Jessop |
| Royal Free Hospital, Gray's Inn Road, W.C. | M., F., 9 | Mr. Mackinlay |

